# ABCC1 promotes GSH-dependent iron transport and resistance to Fe(II) and Cu(II) chelators

**DOI:** 10.1007/s10534-025-00736-z

**Published:** 2025-08-25

**Authors:** Anikó Gaál, Lúcia Torma, Éva Bakos, Katalin Német, Peter Kregsamer, Christina Streli, Miklós Péter Mohai, Elina Üveges, Julia H Bormio Nunes, Petra Heffeter, Gergely Szakács, Norbert Szoboszlai

**Affiliations:** 1 Biological Nanochemistry Research Group, Institute of Materials and Environmental Chemistry, HUN-REN Research Centre for Natural Sciences, Budapest, Hungary; 2 Doctoral School of Pharmaceutical Sciences, Semmelweis University, Budapest, Hungary; 3 Institute of Molecular Life Sciences, HUN-REN Research Centre for Natural Sciences, Budapest, Hungary; 4 Creative Cell Ltd, Budapest, Hungary; 5 Institute of Atomic and Subatomic Physics, Vienna University of Technology, Vienna, Austria; 6 X-Ray Center TU Wien, Vienna, Austria; 7 Institute of Materials and Environmental Chemistry, HUN-REN Research Centre for Natural Sciences, Budapest, Hungary; 8 Center for Cancer Research, Medical University of Vienna, Vienna, Austria; 9 Institute of Inorganic Chemistry, Faculty of Chemistry, University of Vienna, Vienna, Austria; 10 Integrative Health and Environmental Analysis Research Laboratory, Institute of Chemistry, ELTE Eötvös Loránd University, Budapest, Hungary

**Keywords:** ABC transporters, Multidrug resistance, Anticancer chelators, Collateral sensitivity, Iron transport

## Abstract

Therapy resistance is a major challenge in cancer treatment, with multidrug resistance (MDR) being a well-characterized phenomenon wherein cancer cells develop resistance not only to the administered drug but also to structurally diverse compounds. Metal chelators, including quinolines and thiosemicarbazones, have shown broad antitumor activity and potential in overcoming MDR by modulating metal metabolism. However, predicting whether chelators increase or decrease toxicity in MDR cells remains complex, especially as chelator-metal complexes may become substrates for ATP-binding cassette (ABC) transporters, leading to resistance or hypersensitivity. In this study, we developed cell lines with comparable expression levels of the three major MDR ABC transporters (ABCB1, ABCC1, and ABCG2). Our findings demonstrated distinct transporter-specific influences on chelator toxicity. Cells expressing ABCC1 displayed significant resistance to thiosemicarbazones (Dp44mT, COTI-2, DpC) in both their metal-free and metal-bound forms. Vesicular transport assays further demonstrated that ABCC1 actively transports the Fe-GSH complex, formed under physiological glutathione levels, indicating its role in regulating the labile iron pool and reducing intracellular iron toxicity. These findings underscore the importance of transporter-chelator interactions in shaping drug resistance and sensitivity and highlight the intricate roles of ABC transporters in modulating chelator activity.

**Supplementary Information:**

The online version contains supplementary material available at 10.1007/s10534-025-00736-z.

## Introduction

Although a variety of anticancer therapies with diverse mechanisms are available, therapy resistance remains a significant challenge(Housman et al. 2014). One well-documented form of resistance is multidrug resistance (MDR)(Gottesman et al. 2002), wherein cancer cells acquire resistance not only to the administered drug but also to a wide range of structurally dissimilar compounds. A key mechanism contributing to MDR involves the energy-dependent efflux of drugs from cancer cells, leading to decreased intracellular drug concentrations and reduced efficacy. This active transport process is mediated by a family of proteins known as ATP-binding cassette (ABC) transporters. In particular, three human ABC transporters have been implicated in clinical MDR, belonging to the ABCB (ABCB1/MDR1/P-glycoprotein), ABCC (ABCC1/MRP1), and ABCG (ABCG2/MXR/BCRP) subfamilies(Robey et al. 2018). Extensive clinical and experimental research has shown that these pumps recognize a broad array of drug substrates(Gottesman et al. 2002). ABCB1 primarily exports large hydrophobic molecules, whereas ABCC1 and ABCG2 can transport both hydrophobic drugs and larger anionic drug conjugates. Together, the overlapping substrate specificity of the three MDR-ABC transporters forms a powerful shield against drugs in MDR cancer cells(Robey et al. 2018).

The broad antitumor activity exhibited by various classes of metal chelator structures, including quinolines, thiosemicarbazones, and others, underscores their potential in overcoming resistance to established antitumor agents(Whitnall et al. 2006; Wu et al. 2007; Jansson et al. 2010; Pape et al. 2018, 2021; Cserepes et al. 2020). Among these, thiosemicarbazone structures have emerged as particularly promising candidates for overcoming MDR in cancer treatment. In the context of thiosemicarbazones, essential metal ions such as iron, copper, and zinc play significant roles in their mode of action. While the primary focus has been on iron chelation, several studies suggest that the enhanced efficacy of thiosemicarbazones may also be linked to the chelation of other essential metal ions, especially copper (Cu)(Lovejoy et al. 2011; Gaál et al. 2014, 2018a, b). Recent studies have shed light on the role of copper(II) in the anticancer activity of thiosemicarbazones, such as COTI-2(Bormio Nunes et al. 2020). In the presence of copper(II), COTI-2 forms stable complexes with glutathione (GSH), termed GSH-Cu(II) complexes.

ABCC1 exhibits broad substrate specificity(Bakos and Homolya 2007), primarily transporting amphipathic, organic anions, including GSH-, glucuronide- or sulfate-conjugates(Schinkel and Jonker 2012). It plays a pivotal role in the transport of metal complexes, particularly in the context of detoxification processes(Wortelboer et al. 2008). GSH, a tripeptide composed of glutamate, cysteine, and glycine, facilitates the trafficking and export of various cellular metal agents by ABCC1(Helbig et al. 2008). The sulfur atom in reduced GSH exhibits a high affinity for softer metal cations and metal complexes, including mercury, arsenic, antimony, cadmium, lead, and metal-based chemotherapeutic agents such as cisplatin. This high-affinity binding enables the detoxification of metals and metal complexes through reduction, sequestration, and subsequent efflux(Pearson and Cowan 2021).

A small modification of the chemical structure can fundamentally change the toxicity of chelators in MDR cells(Heffeter et al. 2019). For example, the thiosemicarbazone Triapine shows reduced toxicity in ABCB1-expressing cells(Miklos et al. 2015), while its N-terminal dimethylation results in recognition by ABCC1(Heffeter et al. 2012). Certain thiosemicarbazones, such as NSC73306 and its derivatives, show increased toxicity against ABCB1-expressing MDR cells(Ludwig et al. 2006; Hall et al. 2009). The identification of MDR-selective compounds, particularly those with metal chelator structures, highlights a fascinating aspect of overcoming MDR in cancer cells(Szakács et al. 2014). These compounds exhibit selectivity towards multidrug-resistant cells and often possess chemical groups such as thiosemicarbazones, 8-hydroxyquinoline derivatives, and 1,10-phenanthrolines(Szakács et al. 2014). The characteristic feature of these compounds is their ability to form stable complexes with metal ions such as iron(II), iron(III), copper(II), and zinc. The ability of these compounds to chelate metal ions highlights their potential as anticancer agents and also provides valuable insight into the complex interplay between metal metabolism and drug resistance in cancer cells(Szakács et al. 2014).

It is difficult to predict whether a chelator will exhibit increased or decreased toxicity in a MDR cell line. Studies on resistance development against anticancer thiosemicarbazones have underscored the importance of essential metal ions, including Fe(II), Cu(II), and Zn(II)(Pósa et al. 2022). However, the impact of other physiologically relevant metal ions on the anticancer activity and acquired resistance profile of thiosemicarbazones remains largely unknown(Ding and Lind 2009). Given the lack of comparative studies on the interaction between chelators and efflux transporters, our aim was to establish a comprehensive experimental model to evaluate and compare the effects of the three major MDR ABC transporters on the toxicity of metal chelators. Selection of the compounds was guided by the following criteria: (1) cytotoxicity with reported IC₅₀ values across a broad range of cell types; (2) the availability of well-characterized copper and iron complexes documented in the literature; (3) increased toxicity in the presence of copper; and (4) preferential inclusion of compounds featuring chemical scaffolds associated with collateral sensitivity, such as thiosemicarbazones, phenanthrolines, or 8-hydroxyquinolines.

## Results

### Effect of MDR ABC transporters on the toxicity of anticancer chelator ligands

**Fig. 1:**
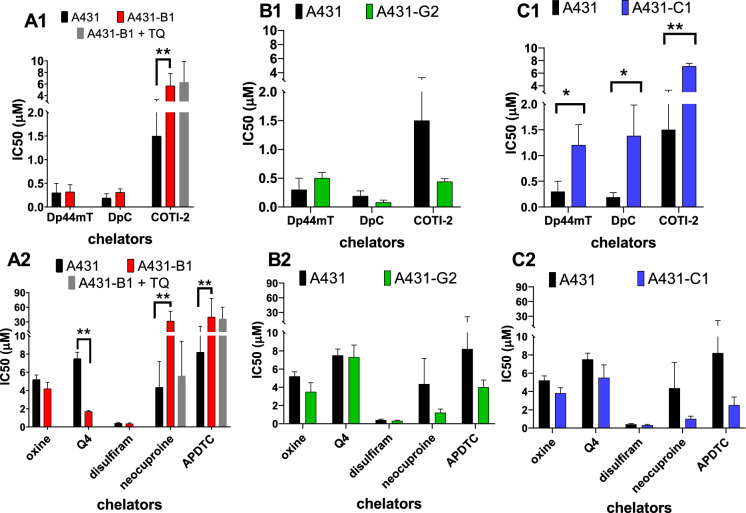
IC_50_ values (µM, 72 h) of the different chelators in A431 parental and MDR A431 cells expressing ABCB1 (A1-A2), ABCG2 (B1-B2) or ABCC1 (C1-C2). ^*^, versus parental, p < 0.05; ^**^, versus parental, p < 0.01

We studied the toxicity of various chelators in the presence of 2 µM Cu(II) or 10 µM Fe(II) ions against A431 cells and their multidrug-resistant derivatives overexpressing ABCB1, ABCG2, or ABCC1. The metal concentrations used in this study were previously determined to be non-toxic(Mihucz et al. 2016; Gaál et al. 2018a). In absence of metal ions, the toxicity of the studied chelators in control cells encompasses a wide range; compounds with a thiosemicarbazone structure exhibited an order of magnitude higher toxicity than the other compounds (Table 1). In order to compare the combined effect of metals and chelators in a comprehensive in vitro model, we engineered multidrug-resistant A431 cells overexpressing ABCB1/P-glycoprotein, ABCG2 or ABCC1/MRP1 (Fig S1, Fig S2, Fig S3). Overexpression of the MDR ABC transporters resulted in significant changes of the toxicity of the studied chelators (Fig. 1). In A431 cells, ABCB1 conferred resistance to neocuproine, APDTC and COTI-2, but did not alter the toxicity of Dp44mT and DpC. Surprisingly, resistance of A431-B1 cells to APDTC and COTI-2 was not abrogated in the presence of tariquidar, which fully abolished resistance to doxorubicin. Dp44mT, shown earlier to selectively target multidrug-resistant ABCB1 expressing cells(Jansson et al. 2015), did not prove to be more toxic to A431 cells overexpressing ABCB1, in line with our earlier report establishing the lack of robust MDR-selective toxicity of this compound(Füredi et al. 2017). In contrast, A431-B1 cells showed increased sensitivity to Q4, confirming the paradoxical hypersensitivity of MDR cells against 8-hydroxyquinoline-derived Mannich bases(Cserepes et al. 2020). Interestingly, overexpresion of ABCG2 did not change the sensitivity of the cells, whereas ABCC1 conferred resistance to the 3 thiosemicarbazones, without any influence on the toxicity of the other compounds.

**Table 1:** IC_50_ values (µM) measured after a 72-h treatment of A431parental cells

	A431-parental		
	w/o metal ions	with Fe(II)	with Cu(II)
Dp44mT	0.3 ± 0.2	0.22 ± 0.13	0.11 ± 0.05
DpC	0.19 ± 0.09	0.19 ± 0.14	0.06 ± 0.03
COTI-2	1.5 ± 1.8	0.26 ± 0.15	0.100 ± 0.035
oxine	5.2 ± 0.5	3.9 ± 1.0	2.7 ± 1.0
Q4	7.5 ± 0.7	8 ± 1	3.40 ± 0.58
disulfiram	0.4 ± 0.1	0.34 ± 0.10	0.29 ± 0.10
neocuproine	4.4 ± 2.8	1.3 ± 0.3	0.29 ± 0.05
APDTC	8.2 ± 13.0	3.5 ± 0.8	1.58 ± 0.30

### Effect of Cu(II) ions on toxicity

**Fig. 2:**
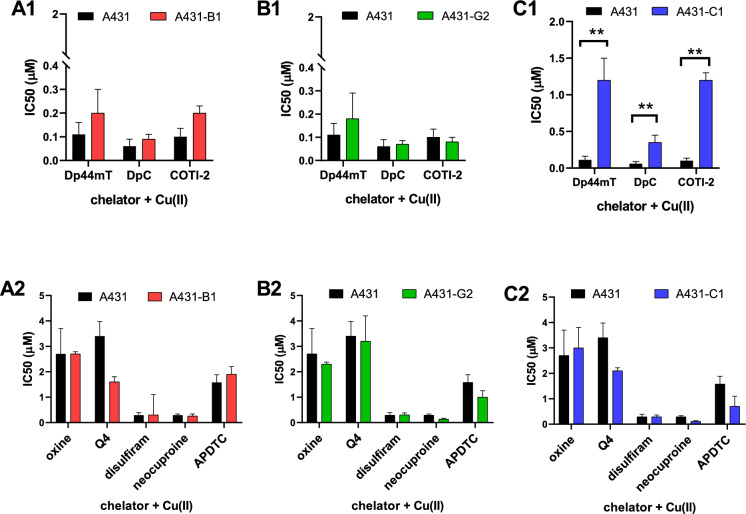
IC_50_ values (µM, for 72 h) of the different chelators in the presence of 2 µM Cu(II) ions in A431 parental and MDR A431 cells expressing ABCB1 (A1-A2), ABCG2 (B1-B2) or ABCC1 (C1-C2). ^*^, versus parental, p < 0.05; ^**^, versus parental, p < 0.01

All of the chelators tested form a complex with copper, thus their toxicity is expected to increase in the presence of Cu(II) ions. Indeed, addition of excess Cu(II) ions significantly enhanced the in vitro toxicity of the studied chelators in A431 cells, resulting in approximately a three-fold elevation. However, the "copper effect" varied among individual chelators, with neocuproine showing a particularly pronounced response, exhibiting a ten-fold increase in toxicity (Table 1, Fig. 2). The presence of Cu(II) ions blunted the ability of ABCB1 to protect A431 cells (relative resistance, determined as the ratio of IC50 values measured in ABCB1-positive MDR and parental cells (RR): 7.4, 4.9, 3.8 vs RR(Cu): 0.9, 1.2, 2.0 for neocuproine, APDTC and COTI-2, respectively). In contrast, the presence of Cu(II) ion did not influence ABCC1-mediated resistance to the thiosemicarbazone compounds (Table 2). As observed without the addition of metals, the expression of ABCG2 did not influence the toxicity of the compounds.

**Table 2:** Relative resistance (RR) of the studied thiosemicarbazone chelators on the A431-C1 cells compared to A431 parental cells

	RR		
	without metals	in the presence of Cu	in the presence of Fe
Dp44mT	4.0	10.9	5.2
DpC	7.3	5.8	8.9
COTI-2	15.8	12.0	61.5

### Effect of Fe(II) ions on toxicity

**Fig. 3:**
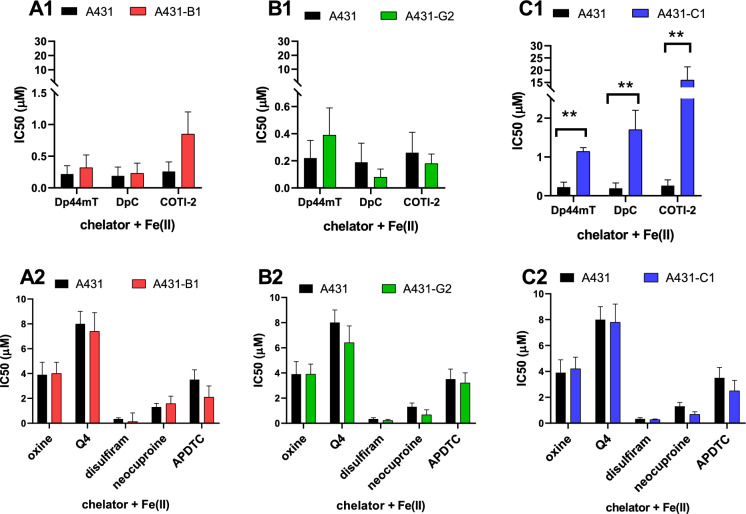
IC_50_ values (µM, for 72 h) of the different chelators in the presence of 10 µM Fe(II) in A431 parental and MDR A431 cells expressing ABCB1 (A 1-A2), ABCG2 (B1-B2) or ABCC1 (C1-C2). ^*^, versus parental, p < 0.05; ^**^, versus parental, p < 0.01

**Fig. 4:**
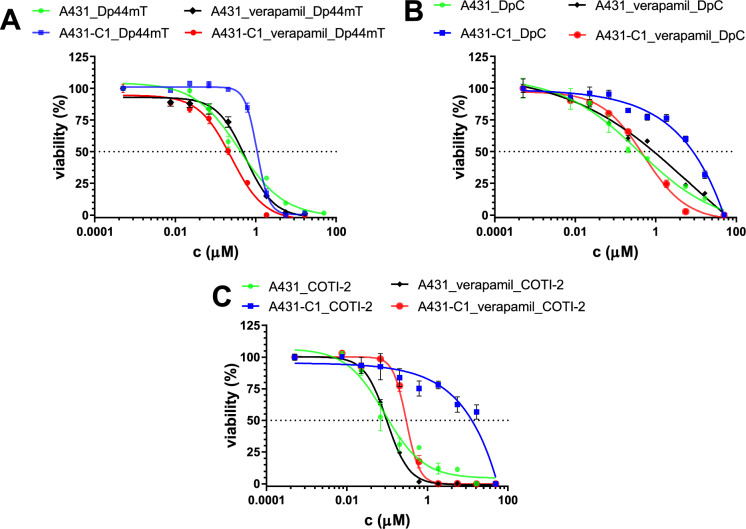
Effect of verapamil on the toxicity of the studied thiosemicarbazones in the presence of iron. Sigmoidal dose–response curves showing the toxicity of Dp44mT (**A**), DpC (**B**) and COTI-2 (**C**) in the presence of Fe(II) ions and verapamil (10 µM) in A431 parental and A431-C1 cells

Excess Fe(II) did not significantly change the toxicity of the studied chelators (Table 1). ABCB1-mediated resistance to COTI-2, neocuproine and APDTC was eliminated in the presence of Fe(II). Interestingly, the presence of iron rescued A431-B1 from the MDR-selective toxicity of Q4 (RR: 0.23 vs RR(Fe): 0.93) (Fig. 3) (Cserepes et al. 2020). While ABCG2 did not influence the toxicity of the compounds, the presence of iron enhanced the protective effect of ABCC1 in A431 cells against DpC (RR: 7.3 vs RR(Fe): 8.9) and significantly increased it in the case of COTI-2 (RR: 15.8 vs RR(Fe): 61.5; p = 0,047) (Table 2). Resistance of A431-ABCC1 cells was eliminated in the presence of the ABCC1-inhibitor verapamil (Fig. 4), and MK-571 (Fig S4) indicating that ABCC1-mediated resistance to the studied TSCs is based on the efflux of iron complexes.

To confirm that the data generated in the A431 model are translatable to other cell systems, we repeated key experiments in additional established resistance models. Puzzled by the lack of effect of tariquidar on the resistance of A431-B1 cells to COTI-2, we next investigated the role of ABCB1 in mediating resistance using the parental KB-3–1 cells and their ABCB1-overexpressing subline, KBC-1. Notably, KBC-1 cells also exhibited approximately fivefold resistance to COTI-2, which was fully abolished in the presence of either metal (Fig S5).

**Fig. 5:**
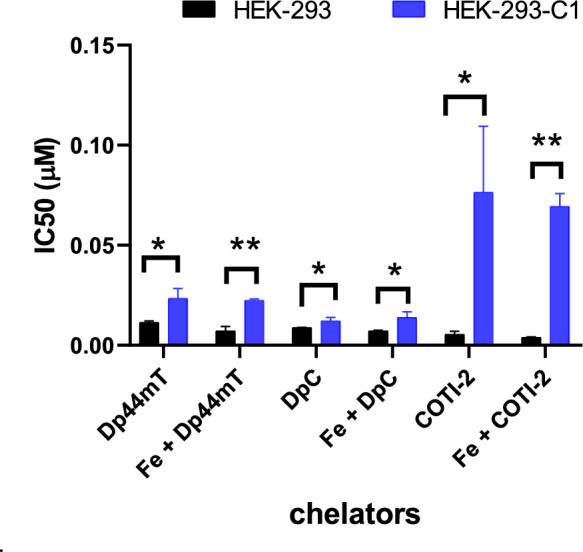
IC_50_ values (µM, for 72 h) of the thiosemicarbazone chelators in the presence and the absence of Fe(II) in HEK-293 parental and ABCC1-expressing cells

The role of ABCC1 in resistance to the studied thiosemicarbazones was further evaluated using parental HEK-293 cells and their ABCC1-transfected derivative, HEK-293-C1. Compared to A431 cells, parental HEK cells exhibited significantly greater sensitivity; however, ABCC1-mediated resistance remained detectable (Fig. 5). Consistent with previous observations, this resistance was reversed by verapamil (Fig S6).

**Fig. 6:**
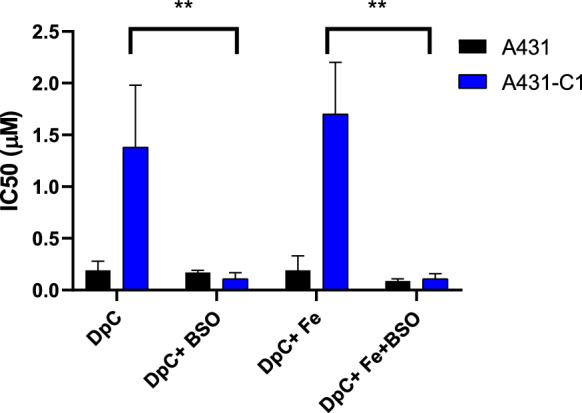
IC_50_ values (µM, at 72 h) of the thiosemicarbazone chelator DpC in the presence and the absence of Fe(II) and BSO in parental and ABCC1-expressing A431cells

To substantiate the role of GSH in ABCC1-mediated resistance, cells were preincubated with buthionine-sulfoximine (BSO) (1 µM, 18 h) prior to DpC treatment. While BSO had no affect on the sensitivity of parental cells, depletion of GSH effectivey abrogated ABCC1-mediated resistance to DpC, both in in the presence and absence of iron (Fig. 6).

**Fig. 7:**
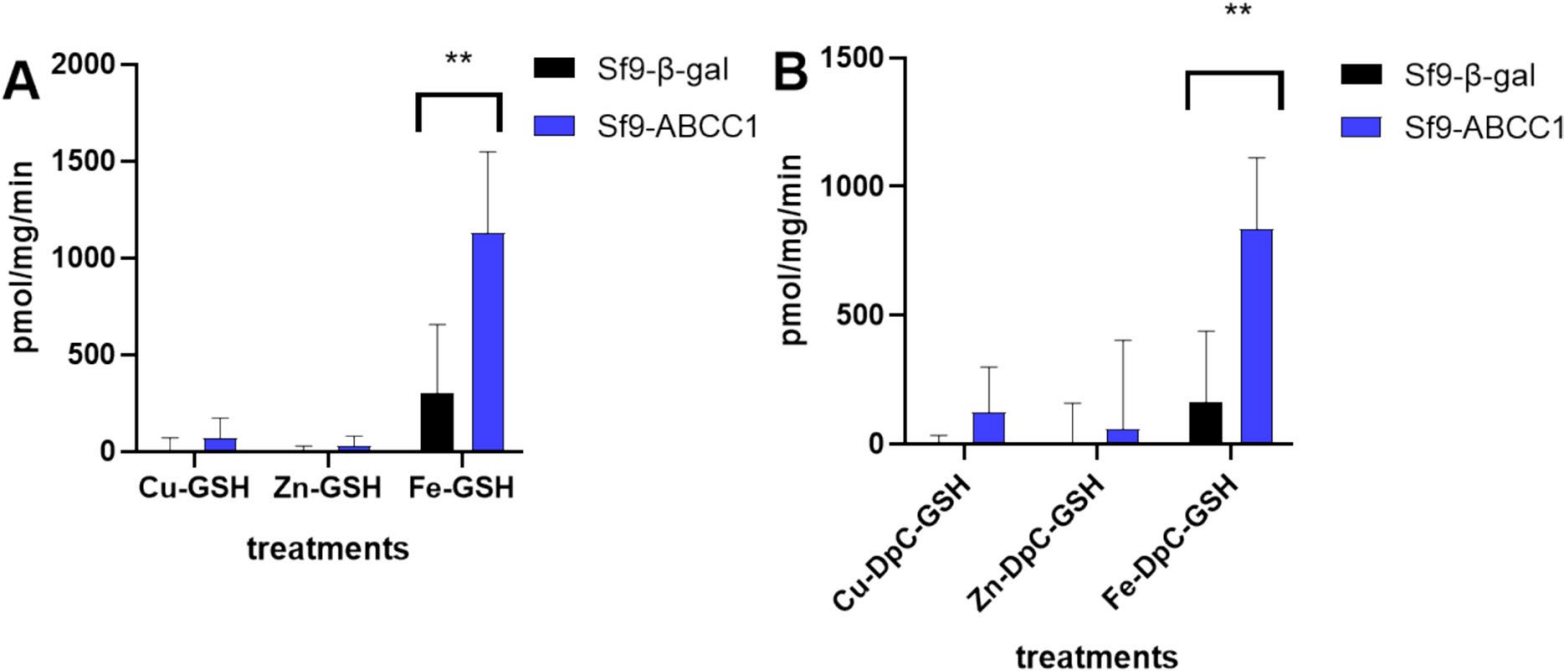
Uptake of Cu, Zn or Fe into Sf9 membrane vesicles containing ABCC1 or β-galactosidase. **A** Accumulation of 25 µM Cu, Zn or Fe in the presence of GSH (5 mM) **B** Accumulation of 25 µM Cu, Zn or Fe after complexed with DpC (25 µM) in the presence of GSH (5 mM). ATP-dependent uptake was calculated by subtracting the values obtained in the presence of 4 mM AMP from those in the presence of 4 mM ATP. Each data point represents the average of triplicate experiments

Reports have shown that thiosemicarbazones forming stable copper(II)-GSH adducts are recognized by ABCC1 for efflux(Bormio Nunes et al. 2020). In the next set of experiments, we performed vesicular transport measurements to determine the nature of transported complexes. In particular, we evaluated the ATP-dependent transport of metals (Fe, Cu, Zn) or DpC in the presence of GSH into inverted Sf9 membrane vesicles expressing human ABCC1. Rather than following the accumulation of DpC, we measured vesicular metal content by ICP-MS and TXRF analysis. ATP-dependent transport of Fe, Cu, and Zn was similar to the levels observed in the presence of AMP or control Sf9 membrane vesicles. ABCC1-mediated accumulation of copper and zinc could not be demonstrated even in the presence of GSH or DpC (Fig. 7a, b). However, ATP-dependent accumulation of iron could be readily demonstrated when the Fe-DpC-GSH complex was added to the vesicles. Strikingly, accumulation of iron was also detected without the presence of the chelator, suggesting that ABCC1 transports Fe-GSH complexes with an activity of 1130 pmol/mg/l (Fig. 7a). The presence of verapamil completely abrogated the ABCC1-mediated transport of Fe-GSH (Fig S7).

## Discussion

Disruption of metal ion balance by chelators can affect cellular processes critical for cancer cell survival and proliferation, contributing to their anticancer effects. Conversely, metal ions can form stable complexes with chelators, altering their chemical properties and affecting their ability to interact with cellular targets. Metal-chelator complexes may also become substrates for ABC transporters, resulting in resistance or collateral sensitivity. In this paper, we systematically investigated the impact of copper and iron on the toxicity of various anticancer chelators. The selected chelating agents represent key compounds of recent interest, particularly concerning anticancer therapy and metal-chelator toxicity. To assess the influence of excess Fe(II) and Cu(II) on chelator toxicity, a fixed amount of each metal was added to the cells before incubation with increasing concentrations of the chelators. In our previous study, we demonstrated that the uptake of inorganic iron is substantial and unaffected by TFR1 expression. Additionally, the introduction of low-concentration iron markedly increased intracellular iron levels(Mihucz et al. 2016). In contrast, the cellular absorption of small amounts of free copper is inhibited in the presence of cellular media; rapid entry into cells is facilitated by complex formation with chelators(Gaál et al. 2018a).

The results reveal that thiosemicarbazones exhibit pronounced cytotoxicity across all tested cell lines. Notably, copper significantly enhances toxicity, consistent with the well-established effect of Cu(II) ions on chelator-induced cytotoxicity(Gaál et al. 2018a, b). Complexation with Fe can increase or decrease the IC_50_ value. Increased toxicity in the presence of Fe can be attributed to the redox properties of the iron-complex system. This redox active metal can undergo redox cycling reactions and can generate reactive radicals leading to oxidative damage. Conversely, decreased cytotoxicity may be explained by the ligand’s reduced intracellular access to iron. Alternatively, the preformed complex may block the chelation of iron by a sensitive cell component involved in proliferation, such as ribonucleotide reductase (Richardson et al. 2006). In the case of copper, increased toxicity is believed to be linked to a coordinated sequence of events (lysosome rupture and/or the reduction of the GSH/GSSG ratio) that induce significant cytotoxicity(Lovejoy et al. 2011; Gaál et al. 2018a).

Resistance to heavy metals is known to be linked to the overexpression of ABCC transporters(Vernhet et al. 1999). In particular, cellular detoxification by ABCC1 and ABCC2 is mediated through the efflux of metal ions such as mercury(Granitzer et al. 2020) or arsenic(Leslie et al. 2004; Csanaky and Gregus 2005) in the form of metal–GSH complexes(Pearson and Cowan 2021). Furthermore, it has been shown that ABCC1 confers resistance to non-metals such as arsenic in association with GSH (Leslie et al. 2004; Leslie 2012). Cadmium-GSH complex efflux proteins have been identified in animal cells(Rakvács et al. 2019), underscoring the importance of ABC transporters in the detoxification pathway for cadmium(Sigel et al. 2013). Bismuth, known for its high affinity for GSH, forms Bi-GSH adducts that are transported into vesicles via ABCC1, as observed in human HK-2 cells (Hong et al. 2015).

In this study, we developed a comprehensive in vitro tool to evaluate the role of efflux mechanisms by achieving comparable expression levels of the three major MDR ABC transporters within the same cell line. This integrated analysis revealed that the three MDR transporters have different effects on the toxicity of the studied chelators. ABCG2 does not influence the toxicity of the ligands or their metal complexes, and the role of ABCB1 could not be unanbiogously confirmed. Intriguingly, functional expression of ABCB1/P-glycoprotein induces hypersensitivity, rather than resistance, to the 8-hydroxyquinoline-derived Mannich base Q4. Collateral sensitivity of MDR cells to Q4 has been shown to result from the inadvertent depletion of intracellular iron pools, caused by the ABCB1-mediated efflux of Q4-iron complexes. Consistent with this mechanism, the hypersensitivity of A431-B1 cells is reversed when excess iron is provided, restoring normal iron levels(Cserepes et al. 2020) (Szakács et al. 2014). Interestingly, our results reveal that MDR cells overexpressing ABCC1 or ABCG2 are not hypersensitive to Q4, likely because the Q4-iron complex is not recognized by these transporters.

Significantly, ABCC1 cells show significant resistance to thiosemicarbazones (Dp44mT, COTI-2, DpC) in the absence of metals. This resistance persists in the presence of both copper and iron, with iron significantly increasing the relative resistance values in all instances. In a recent study, copper and iron complexes of COTI-2 were synthesized and evaluated for their anticancer effects and susceptibility to resistance compared to the metal-free thiosemicarbazone(Bormio Nunes et al. 2020). COTI-2-resistant SW480 cells were found to overexpress ABCC1. Our data indicate that substrate recognition by ABCC1 also encompasses further thiosemicarbazones beyond COTI-2. Pre-synthesized iron complexes are not substrates for ABCC1 per se (Bormio Nunes et al. 2020), but we found that by increasing iron levels, the iron complexes are also recognized by ABCC1.

Using BSO, we demonstrated that the observed resistance—both in the absence and presence of iron—is clearly dependent on intracellular glutathione (GSH) levels. The biochemistry of iron and GSH is closely related, as the Fe-GSH complex is regarded as a primary component of the cellular iron pool (Hider and Kong 2011). The log K value of 5.1 was obtained for Fe-GSH complex which, since the concentration of GSH is relatively high, is sufficient for it to be the most important form of iron in the pool. Moreover it was shown, that the maturation of the cytosolic iron-sulfur proteins require GSH (Sipos et al. 2002). ABCC1 plays a key role in GSH homeostasis (Hider et al. 2021), and the ABCC1-mediated transport of metal-GSH complexes higlights the intricate interplay between metal metabolism and cellular defense mechanisms (Munoz et al. 2007). The vesicular transport assays presented in this study provide indirect evidence that the Fe-DpC complex, in the presence of GSH, is a substrate of ABCC1. These results suggest that ABCC1 recognizes the ternary complex (GSH-Fe(II)-chelator); however, in the case of thiosemicarbazones this complex is usually not stable enough in the presence of typical GSH concentrations (Santoro et al. 2019).

The transport of GSH and its substrates by ABCC1 involves multiple mechanisms, involving transport of GSH, GSH-conjugates, GSH-stimulated transport of substrates or GSSG efflux (Cole and Deeley 2006). Watts et al. (2006) reported that ABCC1 mediates the efflux of an Fe-GSH-NO complex, analogous to the transport of the As(GS)_3_ complex (Leslie et al. 2004). In addition, ABC transporters may also be responsible for the transport of GSH complexed iron-sulfur clusters (Qi et al. 2014). Notably, the vesicular transport assays demonstrate that ABCC1-dependent iron accumulation occurs in the presence of GSH, even in the absence of an exogenous chelator. Since molecules such as LTC4, in which an organic moiety is covalently linked to GSH, are efficiently transported by ABCC1, the most straightforward interpretation of our results is that ABCC1 transports Fe-GSH complexes. However, it is also possible that the Fe-GSH complex dissociates, and GSH is either co-transported, or acts as an allosteric modulator, facilitating iron transport. This observation has significant physiological implications, suggesting that ABCC1 may regulate the labile iron pool, mitigate elevated intracellular iron levels, and contribute to defense against iron toxicity.

## Materials and methods

### Chemicals

Throughout the experiments, deionized Milli-Q (Millipore, Molsheim, France) water with a resistivity of 18.2 MΩ·cm was used. All of the chemicals were of analytical grade. The various chelator-like structures (Supplementary Table 1) were obtained from Sigma-Aldrich (St. Louis, MO, USA), except COTI-2, which was purchased from Selleck Chemicals GmbH (Cologne, Germany). 5-chloro-7-((2-fluorobenzylamino)methyl)quinolin-8-ol (Q4) was previously synthesized and characterized (Pape et al. 2018). L-Buthionine-sulfoximine (BSO), metal salts (iron(II) sulfate and copper(II) sulfate) 5-(3-(2-(7-chloroquinolin-2-yl)ethenyl)phenyl)-8-dimethylcarbamyl-4,6-dithiaoctanoic acid sodium salt hydrate (MK-571), L-glutathione (GSH) and calcein acetoxymethyl ester (Calcein-AM) were purchased from Sigma-Aldrich. Metal salts were freshly dissolved in aqueous solutions, while the chelators were dissolved in DMSO (Sigma-Aldrich). The synthesized iron and copper complexes of COTI-2 were prepared at the Institute of Inorganic Chemistry of the University of Vienna (Vienna, Austria) (Bormio Nunes et al. 2020).

## In vitro experiments

### Cell lines

The A431 human epidermoid carcinoma cell line and the human embryonic kidney-derived HEK-293 cells were obtained from ATCC (LGC Standards GmbH, Wesel, Germany). The epidermal carcinoma-derived human cell line KB-3–1 and its ABCB1-overexpressing subline KBC-1 were generously donated by Dr. Shen, Bethesda, USA (Shen et al. 1986).

### Establishment of ABCC1-expressing cell lines

Cells overexpressing ABCB1 or ABCG2 cells were reported earlier (Nerada et al. 2016). ABCC1-expressing cell lines were established by retroviral transduction. The bicistronic, murine leukemia virus-based vector SPsLdS (Bartlett et al. 1998), containing the cDNAs of gp91phox NADPH oxidase subunit and neomycin resistance gene, was kindly provided by Manuel Grez (Department of Molecular Virology, Georg-Speyer-Haus, Frankfurt am Main, Germany). The bicistronic expression cassette is under the control of a 5’-LTR PCMV (PCC4 embryonal carcinoma cell-passaged myeloproliferative sarcoma virus) promoter and a 3’-LTR SFFV (spleen focus forming virus) enhancer. The cDNA of gp91phox was excised from the SPsLdS vector and ABCC1 cDNA (GenBank accession No. L05628.1) was inserted into the acceptor vector. Phoenix-Eco (ΦNX-Eco) was kindly provided by G. Nolan (Stanford Univ., Stanford, CA) and transfected with vector containing ABCC1 using polyethyleneimine (PEI) (Boussif et al. 1995). 72 h after transfection the supernatant of ΦNX-Eco cells was used to transduce PG13 packaging cells (Miller et al. 1991) (ATCC, Rockville, MD). Vector-positive PG13 clones were cultured and supernatants were collected for further transduction of cell lines. A431 and HEK-293 cell-line (both obtained from ATCC) were transduced by spinoculation (1020 × G for 99 min at 32 °C). Cells were incubated at 32 °C for 48–72 h post-transduction.

### Cell culture conditions

Cell lines were cultured in RPMI-1640 or in Dulbecco's Modified Eagle's Medium (DMEM) media according to manufacturer’s instructions. RPMI and DMEM (Sigma Aldrich) were supplemented with 10% FBS (fetal bovine serum, Gibco, purchased from Thermo Fisher Scientific, Waltham, Massachusetts, US), 5 mM glutamine, and 50 unit/mL penicillin and streptomycin (Life Technologies, Waltham, Massachusetts, US). Cell cultures were kept in a humidified incubator at 37 °C, in 5% CO_2_ atmosphere. Washing steps were executed by Dulbecco's Phosphate Buffered Saline solution (DPBS, without Ca^2+^ and Mg^2+^, Gibco, purchased from Thermo Fisher Scientific); cells were trypsinized by the required concentration of 10 × Trypsin–EDTA solution (5.0 g/L porcine trypsin and 2.0 g/L EDTA·4 Na in 0.9% v/v sodium chloride solution). Cells were counted with either a Bürker counting chamber (Marienfeld Superior–Paul Marienfeld GmbH & Co. KG, Lauda-Königshofen, Germany) or TC20 Automated Cell Counter (Bio-Rad Laboratories, Budapest, Hungary) using Trypan Blue (Gibco, purchased from Thermo Fisher Scientific).

### *Evaluation of *in vitro* toxicity*

Cells were seeded into 96-well tissue culture plates (Sarstedt, Newton, USA/Orange, Braine-l’Alleud, Belgium) at a density of 5,000 cells per well/100 µL. The cells were allowed to attach for 12 h. Metal ions and compounds were added to achieve the required final concentration in a final volume of 200 μL per well. The metal ions were added 30 min before the treatment with the chelator series dilution. After incubation for 72 h, the supernatant was removed, and viability was assessed by the PrestoBlue® assay (Life Technologies, Carlsbad, CA, USA), according to the manufacturer’s instructions. The viability of the cells was spectrophotometrically measured (measuring fluorescence, excitation at 544 nm and emission at 590 nm) using an EnSpire microplate reader (Perkin Elmer). The data were normalized to untreated cells. The curves were fitted by GraphPad Prism 8 software using the sigmoidal dose–response (variable slope) model. Curve fit statistics were used to determine IC_50_ values. The IC_50_ data in Figs. 1, 2, 3 can be found in Supplementary Table 2.

### Viability assays on the KB cell model

Cells were plated (2 × 10^4^ cells/mL for KB-3–1 and KBC-1) in 100 µL/well in 96-well plates and allowed to attach overnight. Compounds were dissolved in DMSO (10 mM stock) and further diluted into growth medium (< 1% DMSO in the final concentrations). Compounds were added in 100 µL/well and cells were exposed to the compounds for 72 h at 37 °C and 5% CO_2_. The proportion of viable cells was determined by the MTT assay following the manufacturer’s recommendations (EZ4U, Biomedica, Vienna, Austria).

### Transport assay using Sf9 membrane vesicles

ABCC1 transport function was assayed in isolated Sf9 (Spodoptera frugiperda ovarian cells) membrane vesicles produced in the baculovirus-insect cell system. Virus-infected Sf9 cells were harvested, their membranes were isolated and stored, and the membrane protein concentrations were determined as described, with minor modifications(Bakos et al. 1998, 2000). Control vesicles were prepared from Sf9 cells infected with baculovirus containing β-galactosidase.

Complexes were prepared as follows: 100 mM stock solution of metal ion solutions (FeSO_4_, ZnSO_4_, CuSO_4_) in water, 500 mM GSH stock solution in water and 100 mM stock solution of DpC in DMSO were freshly prepared and diluted to 250 µM metal and/or chelator concentration with or without 50 mM GSH. A few minutes before the experiments, the pH of the obtained solutions was adjusted to 7.4. These solutions were diluted to ten times to the transport buffer solution to get 25 µM metal and 0 or 5 mM GSH solutions. Membrane vesicles (50 µg of protein) were incubated at 37 °C at a final volume of 150 µL for 3 min. The transport assay buffer used was HEPES (50 mM, pH 7.4), KCl (50 mM), MgCl_2_ (6 mM) and contained ATP or AMP (4 mM) with and without GSH (5 mM). The transport was stopped with 1 mL ice-cold TRIS sucrose buffer (50 mM, pH 7.4, 250 mM sucrose) and the vesicules pelleted and washed by centrifugation (16,000 g for 10 min) three times with the same TRIS sucrose buffer. ATP-dependent transport of different compounds was examined based on the determination of intravesicular Fe. Transport in the presence of AMP was subtracted from transport in the presence of ATP and reported as ATP dependent transport.

### Determination of vesicular Fe levels

#### Sample preparation

After the last centrifugation step, the pellets were digested for 24 h at room temperature in 20 μL of 30% H_2_O_2_, and 80 μL of 65% HNO_3_. The samples were directly subjected to TXRF measurement and were diluted to a volume of 5 mL with ultrapure water, if they were subjected to ICP-MS measurement.

#### Inductively coupled plasma mass spectrometry (ICP-MS)

ICP-MS analysis was performed using a Thermo Fisher ICAP-Q inductively coupled plasma mass spectrometer, equipped with an ASX-520 autosampler unit. Kinetic energy discrimination mode was used to eliminate interferences. Three isotopes of iron (54Fe, 56Fe and 57Fe) were used for quantitative analysis. Four calibration points (0; 1; 10; 100 µg/L) were prepared for the quantification, the first being ultrapure (18.2 MΩ*cm) water, the others were diluted from a multielement standard solution (CPA-Chem, 33 elements, each 1000 mg/L in 2% nitric acid), each point containing 1 mL of ultrapure 67% nitric acid in a total volume of 50 mL.

#### Total-reflection X-ray fluorescence (TXRF) analysis

Fe content was determined by the TXRF method, as reported elsewhere(Szoboszlai et al. 2009; Polgári et al. 2011), using an Atomika 803^0C^ TXRF spectrometer (Atomika Instruments GmbH, Oberschleissheim, Munich, Germany). Gallium was used as an internal standard. The stock solution of 1000 mg/L Ga was purchased from Merck (Darmstadt, Germany). The metal data were determined by averaging the data measured by the two instrumental analytical techniques (ICP-MS and TXRF).

#### Western blot and flow cytometric analysis

Immunoblotting was performed after dissolving and sonicating the cells in a disaggregation buffer. ABCC1 was detected with the monoclonal antibody ABCC1 (Novus Biologicals MRPm6 monoclonal antibody). Protein-antibody interaction was determined using the enhanced chemiluminescence technique as described previously(Bakos et al. 1998, 2000)*.*

The function of the ABCC1 transporter was proven by flow cytometric measurements in A431 and HEK cells. The measurements were performed with a CytoFlex flow cytometer (Beckman Coulter Inc, Brea, California, USA) using the CytExpert program. Analysis was performed in FlowJo Software (Becton, Dickinson and Company (BD), Franklin Lakes, New Jersey, USA). Calcein assay was used (Szabó et al. 2018) for the functional characterization of ABCC1, with minor modifications. Verapamil (Sigma-Aldrich, 10 µM concentration) was used as an inhibitor of the ABCC1 transporter.

### Statistical analysis

IC50 values were calculated from normalized dose–response curves, All statistical analyses were performed using GraphPad Prism 8 software (GraphPad Software). Student (two-sided) t test was used to evaluate statistical significance. (^*^, versus control, p < 0.05; ^**^, versus control, p < 0.01). All data represent at least 3 independent experiments.

## Supplementary Information

Below is the link to the electronic supplementary material.

Supplementary file1 (DOCX 479 KB)

## Data Availability

The data presented in this article may be provided by the corresponding author on reasonable request.
