# Supplementary material for: ABCC1 promotes GSH-dependent iron transport and resistance to Fe(II) and Cu(II) chelators
Source: Biometals. 2025 Aug 25;38(6):1881–95. doi: 10.1007/s10534-025-00736-z (PMC12686069; doi:10.1007/s10534-025-00736-z)
Supplement: Supplementary file 1 — Supplementary file1 (DOCX 479 KB) [file 10534_2025_736_MOESM1_ESM.docx]

**Supplementary material**

**ABCC1 promotes GSH-dependent iron transport and resistance to Fe(II) and Cu(II) chelators**

**List of Supplemental Figures and Table**

Figure S1. Flow cytometry analysis of ABCC1 activity evaluated by calcein accumulation. Parental and ABCC1 overexpressing A431 cells were subjected to 0.25 μM Calcein-AM in the absence or presence of the inhibitor of ABCC1 for 30 min. Verapamil (50µM) was used for the inhibition of ABCC1. Fluorescence in live cells was measured by flow cytometry.

Figure S2. Flow cytometry analysis of ABCC1 activity evaluated by calcein accumulation. Parental and ABCC1 overexpressing HEK-293 cells were subjected to 0.25 μM Calcein-AM in the absence or presence of the inhibitor of ABCC1 for 30 min. Verapamil (50µM) was used for the inhibition of ABCC1. Fluorescence in live cells was measured by flow cytometry.

Figure S3. Western blot detection of human ABCC1 expressed in A431, HEK-293 or Sf9 cells. The amounts of total protein loaded onto the gel were the following: A431-ABCC1, A431 parental, HEK-ABCC1, HEK-293 parental l: 20 mg, Sf9-ABCC1, Sf9-b-gal l: 5 mg.

Figure S4. Effect of MK-571 on the toxicity of the studied thiosemicarbazones in the presence of iron. Sigmoidal dose-response curves showing the toxicity of Dp44mT (A), DpC (B) and COTI-2 (C) in the presence of Fe(II) ions and MK-571 (50 µM) in A431 parental and A431-ABCC1 cells.

Figure S5. IC50 values (µM, 72h) of COTI-2, Cu-COTI-2 and Fe-COTI-2 in KB-3-1 parental and MDR KBC-1 cells expressing ABCB1. No statistical significance was found comparing parental and MDR cells in case of the copper and iron complex.

Figure S6. Effect of verapamil on the toxicity of the studied thiosemicarbazones in the presence of iron. Sigmoidal dose-response curves showing the toxicity of Dp44mT (A), DpC (B) and COTI-2 (C) in the presence of Fe(II) ions and verapamil (10 µM) in the HEK-293 parental and HEK-293-ABCC1 cells.

Figure S7. Uptake of Fe into Sf9 membrane vesicles containing ABCC1 or β-galactosidase. Accumulation of 25 µM Fe and 5 mM GSH in the absence or in the presence of verapamil inhibitor. ATP-dependent uptake was calculated by subtracting the values obtained in the presence of 4mM AMP from those in the presence of 4mM ATP. Each data point represents the average of triplicate experiments.

Supplementary Table 1: Investigated chelator-like structures with chemical names. Tested compounds include thiosemicarbazonone, phenanthroline, 8-hydroxyquinoline, dithiocarbamate, and carbothioamide structures. Dp44mT, DpC, COTI-2, oxine, Q4, disulfiram, neocuproine, and APDTC were evolved in the study

Supplementary Table 2: *In vitro* data in Figures 1-3 are given in table form. Figure 1 is shown in Table 2a, Figure 2 in Table 2b and Figure 3 in Table 2c.

Supplementary Figure 1: Flow cytometry analysis of ABCC1 activity evaluated by calcein accumulation. Parental and ABCC1 overexpressing A431 cells were subjected to 0.25 μM Calcein-AM in the absence or presence of the inhibitor of ABCC1 for 30 min. Verapamil (50µM) was used for the inhibition of ABCC1. Fluorescence in live cells was measured by flow cytometry

Supplementary Figure 2: Flow cytometry analysis of ABCC1 activity evaluated by calcein accumulation. Parental and ABCC1 overexpressing HEK-293 cells were subjected to 0.25 μM Calcein-AM in the absence or presence of the inhibitor of ABCC1 for 30 min. Verapamil (50µM) was used for the inhibition of ABCC1. Fluorescence in live cells was measured by flow cytometry


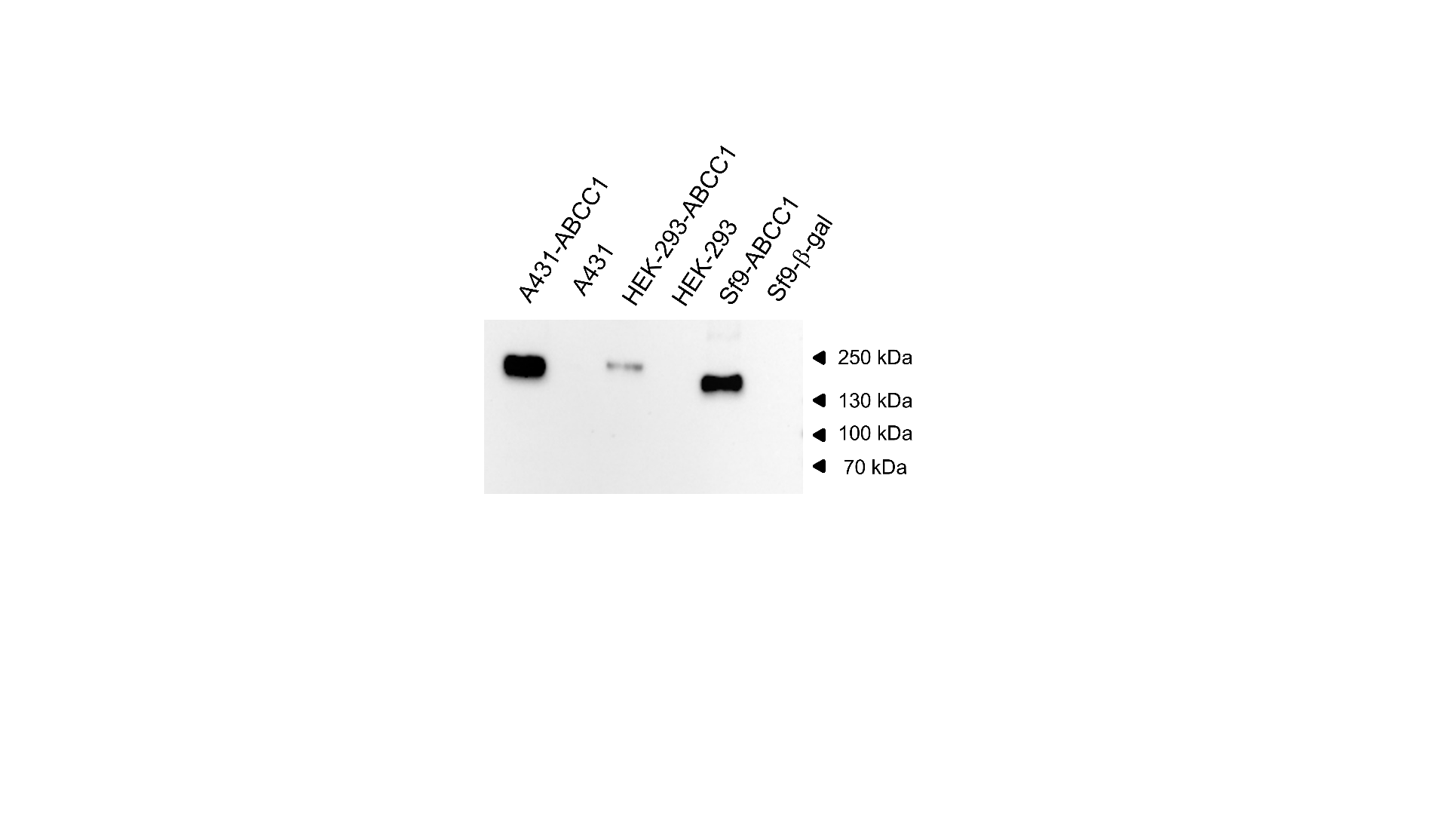


Supplementary Figure 3: Western blot detection of human ABCC1 expressed in A431, HEK-293 or Sf9 cells. The amounts of total protein loaded onto the gel were the following: A431-ABCC1, A431 parental, HEK-ABCC1, HEK-293 parental: 20 µg, Sf9-ABCC1, Sf9 β-gal: 5 µg.


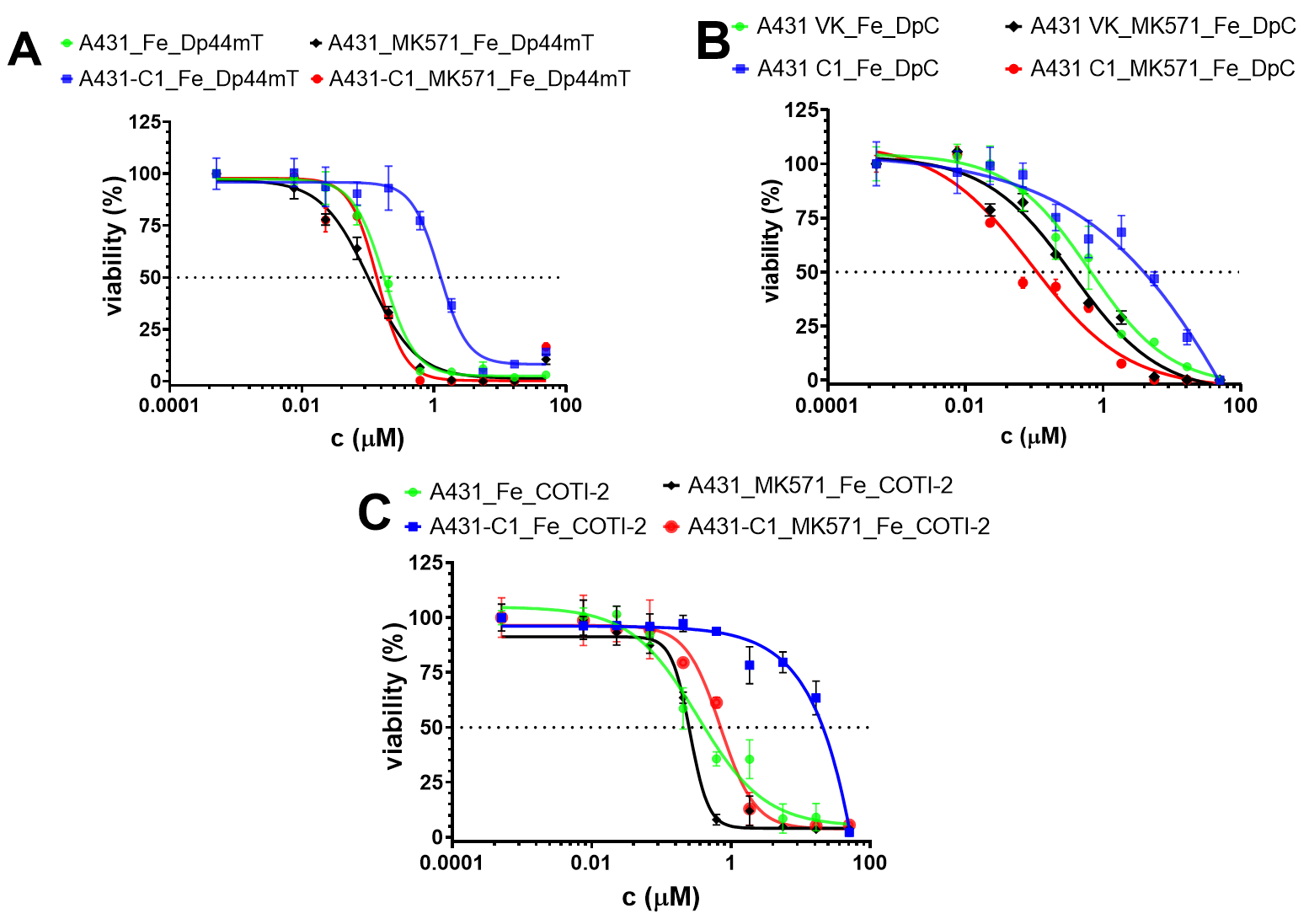


Supplementary Figure 4: Effect of MK571 on the toxicity of the studied thiosemicarbazones in the presence of iron. Sigmoidal dose-response curves showing the toxicity of Dp44mT (A), DpC (B) and COTI-2 (C) in the presence of Fe(II) ions and MK-571 (50 µM) in A431 parental and A431-ABCC1 cells

Supplementary Figure 5: IC50 values (µM, 72h) of COTI-2, Cu-COTI-2 and Fe-COTI-2 in KB-3-1 parental and MDR KBC-1 cells expressing ABCB1. No statistical significance was found comparing parental and MDR cells in case of the copper and iron complex.

Supplementary Figure 6: Effect of verapamil on the toxicity of the studied thiosemicarbazones in the presence of iron. Sigmoidal dose-response curves showing the toxicity of Dp44mT (A), DpC (B) and COTI-2 (C) in the presence of Fe(II) ions and verapamil (10 µM) in the HEK-293 parental and HEK-293-ABCC1 cells

Supplementary Figure 7: Uptake of Fe into Sf9 membrane vesicles containing ABCC1 or β-galactosidase. Accumulation of 25 µM Fe and 5 mM GSH in the absence or in the presence of verapamil inhibitor. ATP-dependent uptake was calculated by subtracting the values obtained in the presence of 4mM AMP from those in the presence of 4mM ATP. Each data point represents the average of triplicate experiments.

**Supplementary Table 1:** Investigated chelator-like structures with chemical names. Tested compounds include thiosemicarbazonone, phenanthroline, 8-hydroxyquinoline, dithiocarbamate, and carbothioamide structures. Dp44mT, DpC, COTI-2, oxine, Q4, disulfiram, neocuproine, and APDTC were evolved in the study

| 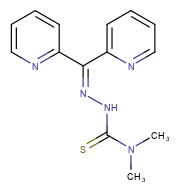 | 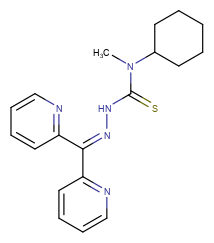 | 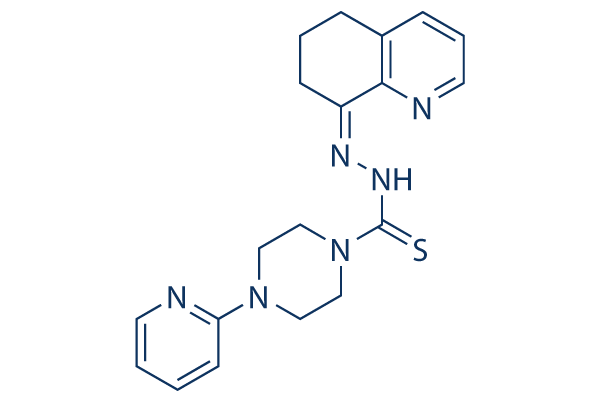 | 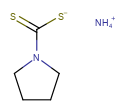 |
| --- | --- | --- | --- |
| **Dp44mT** | **DpC** | **COTI-2** | **APDTC** |
| 2-(Di-2-pyridinylmethylene)-N,N-dimethyl-hydrazinecarbothioamide, Di-2-pyridylketone-4,4,-dimethyl-3-thiosemicarbazone | Di-2-pyridylketone-4-cyclohexyl-4-methyl-3-thiosemicarbazone | N'-(6,7-dihydroquinolin-8(5H)-ylidene)-4-(pyridin-2-yl)piperazine-1-carbothiohydrazide | Ammonium pyrrolidinedithiocarbamate,  1-Pyrrolidinecarbodithioic acid ammonium salt, Ammonium pyrrolidinecarbodithioate, PDC, PDTC |
| 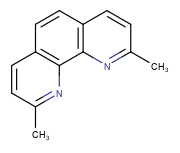 | 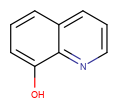 | 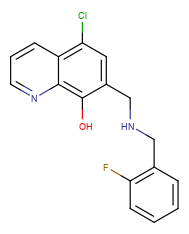 | 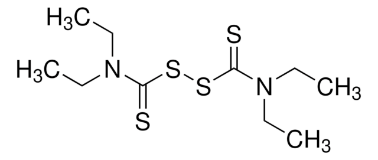 |
| **neocuproine** | **oxine** | **Q4** | **disulfiram** |
| 2,9-Dimethyl-1,10-phenanthroline, DMPHEN | 8-Hydroxyquinoline, 8-Oxychinolin, 8-Quinolinol, Oxine | 5-chloro-7-((2-fluorobenzylamino)methyl)quinolin-8-ol | Tetraethylthiuram disulfide, Bis(diethylthiocarbamoyl) disulfide, Bis(diethylthiocarbamyl) disulfide, Disulfiram, TETD, N,N-diethyl[(diethylcarbamothioyl)disulfanyl]carbothioamide |

**Supplementary Table 2:***In vitro* data in Figures 1-3 are given in table form. Figure 1 is shown in Table 2a, Figure 2 in Table 2b and Figure 3 in Table 2c.

**Supplementary Table 2a**

|  | A431 | A431-B1 | A431-G2 | A431-C1 |
| --- | --- | --- | --- | --- |
|  | IC50 (µM)±SD | IC50 (µM)±SD | IC50 (µM)±SD | IC50 (µM)±SD |
| Dp44mT | 0.30±0.20 | 0.32±0.15 | 0.50±0.10 | 1.20±0.40 |
| DpC | 0.19±0.09 | 0.31±0.07 | 0.08±0.04 | 1.38±0.60 |
| COTI-2 | 1.50±1.80 | 5.70±2.10 | 0.44±0.05 | 7.10±0.46 |
| oxine | 5.20±0.50 | 4.20±0.70 | 3.50±1.00 | 3.80±0.60 |
| Q4 | 7.50±0.70 | 1.70±0.10 | 7.30±1.34 | 5.50±1.4 |
| disulfiram | 0.40±0.10 | 0.35±0.09 | 0.30±0.09 | 0.31±0.09 |
| neocuproine | 4.35±2.80 | 32.0±12.8 | 1.20±0.40 | 1.00±0.30 |
| APDTC | 8.20±13.0 | 40.2±38.0 | 4.00±0.80 | 2.50±0.90 |

**Supplementary Table 2b**

|  | A431 | A431-B1 | A431-G2 | A431-C1 |
| --- | --- | --- | --- | --- |
|  | IC50 (µM)±SD | IC50 (µM)±SD | IC50 (µM)±SD | IC50 (µM)±SD |
| Dp44mT | 0.11±0.05 | 0.20±0.10 | 0.18±0.11 | 1.20±0.30 |
| DpC | 0.06±0.03 | 0.09±0.02 | 0.07±0.02 | 0.35±0.10 |
| COTI-2 | 0.10±0.04 | 0.20±0.03 | 0.08±0.02 | 1.20±0.10 |
| oxine | 2.70±1.00 | 2.70±0.09 | 2.30±0.08 | 3.00±0.80 |
| Q4 | 3.40±0.58 | 1.60±0.20 | 3.20±1.00 | 2.10±0.12 |
| disulfiram | 0.29±0.10 | 0.30±0.80 | 0.30±0.08 | 0.29±0.07 |
| neocuproine | 0.29±0.05 | 0.26±0.08 | 0.14±0.03 | 0.11±0.03 |
| APDTC | 1.58±0.30 | 1.90±0.30 | 1.00±0.25 | 0.70±0.40 |

**Supplementary Table 2c**

|  | A431 | A431-B1 | A431-G2 | A431-C1 |
| --- | --- | --- | --- | --- |
|  | IC50 (µM)±SD | IC50 (µM)±SD | IC50 (µM)±SD | IC50 (µM)±SD |
| Dp44mT | 0.22±0.13 | 0.32±0.20 | 0.39±0.20 | 1.14±0.10 |
| DpC | 0.19±0.14 | 0.23±0.16 | 0.08±0.06 | 1.70±0.50 |
| COTI-2 | 0.26±0.15 | 0.85±0.35 | 0.18±0.07 | 16±5.4 |
| oxine | 3.90±1.00 | 4.00±0.90 | 3.90±0.80 | 4.20±0.90 |
| Q4 | 8.00±1.00 | 7.40±1.50 | 6.40±1.34 | 7.80±1.40 |
| disulfiram | 0.34±0.10 | 0.13±0.70 | 0.22±0.09 | 0.28±0.05 |
| neocuproine | 1.3±0.30 | 1.58±0.60 | 0.67±0.40 | 0.68±0.20 |
| APDTC | 3.5±0.80 | 2.10±0.90 | 3.20±0.80 | 2.50±0.80 |
